# Suicidal ideation across depressive episodes: 9-year longitudinal cohort study

**DOI:** 10.1192/bjo.2023.608

**Published:** 2023-11-20

**Authors:** Liia M. M. Kivelä, Niki Antypa, Eiko I. Fried, Robert Schoevers, Albert M. van Hemert, Brenda W. J. H. Penninx, A. J. Willem van der Does

**Affiliations:** Department of Clinical Psychology, Institute of Psychology, Leiden University, Leiden, The Netherlands; University Center for Psychiatry, University Medical Center Groningen, Groningen, The Netherlands; Department of Psychiatry, Leiden University Medical Center (LUMC), Leiden, The Netherlands; Department of Psychiatry, Amsterdam Public Health Research Institute, Amsterdam UMC, Vrije Universiteit, Amsterdam, The Netherlands; Department of Clinical Psychology, Institute of Psychology, Leiden University, Leiden, The Netherlands; and Leiden University Treatment Center (LUBEC), Leiden, The Netherlands

**Keywords:** Depression, recurrence, suicidal ideation, sleep disturbance, insomnia

## Abstract

**Background:**

Depression is a highly recurrent disorder, with more than 50% of those affected experiencing a subsequent episode. Although there is relatively little stability in symptoms across episodes, some evidence indicates that suicidal ideation may be an exception. However, these findings warrant replication, especially over longer periods and across multiple episodes.

**Aims:**

To assess the relative stability of suicidal ideation in comparison with other non-core depressive symptoms across episodes.

**Method:**

We examined 490 individuals with current major depressive disorder (MDD) at baseline and at least one subsequent episode during 9-year follow-up within the Netherlands Study of Depression and Anxiety (NESDA). The Inventory of Depressive Symptomatology (IDS) was used to assess DSM-5 non-core MDD symptoms (fatigue, appetite/weight change, sleep disturbance, psychomotor disturbance, concentration difficulties, worthlessness/guilt, suicidal ideation) at baseline and 2-, 4-, 6- and 9-year follow-up. We examined consistency in symptom presentation (i.e. whether the symptom met the diagnostic threshold, based on a binary categorisation of the IDS) using kappa (κ) and percentage agreement, and stability in symptom severity using Spearman correlation, based on the continuous IDS scores.

**Results:**

Out of all non-core depressive symptoms, insomnia appeared the most stable across episodes (*r* = 0.55–0.69, κ = 0.31–0.47) and weight decrease the least stable (*r* = 0.03–0.33, κ = 0.06–0.19). For suicidal ideation, correlations across episodes ranged from *r* = 0.36 to *r* = 0.55 and consistency ranged from κ = 0.28 to κ = 0.49.

**Conclusions:**

Suicidal ideation is moderately stable in recurrent depression over 9 years. Contrary to prior reports, however, it does not exhibit substantially more stability than most other non-core symptoms of depression.

Depression is a highly recurrent disorder,^1–3^ but one that can differ greatly in its symptomatic expression from one episode to the next.^4–7^ Suicidal ideation, however, is often a persistent disturbance^8,9^ and specifically tends to re-emerge at times of psychological distress.^10^ With regard to the stability of suicidal ideation in recurrent depression, conflicting findings have been reported. Some studies have shown up to 86% of individuals with a history of suicidal ideation during a previous depressive episode to also report ideation at recurrence.^11^ Further, in this group, suicidal ideation was the only non-core diagnostic criterion symptom (i.e. excluding the core symptoms of depressed mood and anhedonia) that was significantly correlated across episodes, after adjustment for multiple testing.^11^ Among in-patients with current major depressive disorder, anxiety and suicidal ideation were also the most consistent symptoms across episodes, although correlations of symptom severity were weak overall.^7^ The relative stability of suicidal ideation across depressive episodes has been replicated in bipolar disorder.^12^ Other studies, however, have found sleep disturbance and concentration difficulties to be the most stable symptoms,^13^ or all symptoms to have equally low stability.^4–6^ These inconsistencies and low stability estimates may be influenced by both study design (small samples) and psychometric limitations of the measures used to quantify consistency (i.e. the kappa coefficient); kappa can lead to erroneously low estimates in instances of very high (or low) base rates,^14,15^ such as in the case of scoring diagnostic criteria in depressed populations where a minimum number of symptoms is required for a diagnosis. Hence, kappa may be unreliable in cases of too much consistency, especially in small samples. Consequently, the stability of depressive symptoms across episodes warrants replication, especially over longer time intervals and in larger samples. Although symptom severity and expression may vary in many episodic disorders, stability of symptoms has implications for diagnosis and subsequent treatment.

The aim of the present study was to assess the relative stability of non-core depressive symptoms across episodes. In accordance with the DSM-5 major depressive disorder diagnostic criterion A, which requires the presence of at least one of the two core symptoms of depressed mood or anhedonia, the remaining diagnostic criterion symptoms (fatigue, appetite/weight change, sleep disturbance, psychomotor disturbance, concentration difficulties, worthlessness/guilt, suicidal ideation) were considered non-core symptoms.^16,17^ Based on the literature, we expected that suicidal ideation would be the most stable of the non-core diagnostic criterion symptoms. The core symptoms of depressed mood and anhedonia are reported for referential purposes but were excluded from the comparisons (as was done in a previous study^11^), as at least one of the symptoms is always required to be present for a diagnosis. Hence these symptoms by nature are expected to have greater stability than non-core symptoms.

## Method

### Sample

The sample was derived from the Netherlands Study of Depression and Anxiety (NESDA), a large-scale longitudinal cohort study of individuals with and without depression and anxiety disorders (further details are available in the literature^18,19^). All participants provided written informed consent. For the present study, data from the first five assessments up to a 9-year follow-up were included. We included individuals with current major depressive disorder (MDD) at baseline and at least one subsequent depressive episode occurring during the 9-year follow-up.

### Ethics statement

The authors assert that all procedures contributing to this work comply with the ethical standards of the relevant national and institutional committees on human experimentation and with the Helsinki Declaration of 1975, as revised in 2008. All procedures involving human subjects/patients were approved by the Medical Ethical Committee of the VU University Medical Center Amsterdam (2003/183).

### Materials

#### Psychiatric diagnoses

The Composite International Diagnostic Interview (CIDI)^20^ was used to assess the presence of current MDD at baseline and 2-, 4-, 6- and 9-year follow-up. Presence of MDD was defined as fulfilling DSM criteria any time during the past 6 months (CIDI) and having at least mild current depression (Inventory of Depressive Symptomatology IDS ≥ 14) still present at time of measurement; these criteria were used to define the presence of MDD at baseline and recurrence at each follow-up.

#### Depressive symptoms

The Inventory of Depressive Symptomatology – Self-Report version (IDS-SR^21^) was used to assess current depressive symptom severity at baseline and recurrence at 2-, 4-, 6- and 9-year follow-up. The IDS consists of 30 items rated on a four-point Likert scale (0–3) pertaining to depressive symptoms experienced during the past week. For the present study, we used 16 items corresponding to the DSM-5 MDD diagnostic criterion symptoms (i.e. excluding items that are not considered diagnostic symptoms in DSM-5, such as somatisation). (Note: the IDS uses three items to assess insomnia; a mean score of these items was created. All other symptoms were estimated with singular items of the IDS.) To estimate consistency in symptom presentation (i.e. whether a symptom met the diagnostic threshold), the IDS scores were dichotomised based on the Likert scale rating: 0 and 1 were scored as 0 (symptom not present); 2 and 3 were scored as 1 (symptom present). To estimate stability of symptom severity, the continuous IDS scores were used. Descriptives of the IDS items are presented in Table 1.

**Table 1:** Base rates^a^ and descriptives of major depressive disorder symptoms at baseline and at episode recurrence at 2-, 4-, 6- and 9-year follow-up

IDS symptom		Baseline (*n* = 490)	2 years (*n* = 302)	4 years (*n* = 229)	6 years (*n* = 190)	9 years (*n* = 154)
**Core symptoms**						
Depressed mood	Base rate (%)	267 (55%)	141 (47%)	126 (55%)	94 (50%)	81 (53%)
	Mean (s.d.)	1.63 (0.83)	1.48 (0.77)	1.61 (0.79)	1.54 (0.79)	1.59 (0.83)
	Skew	0.02	0.17	–0.01	0.16	0.05
Anhedonia	Base rate (%)	137 (28%)	76 (25%)	61 (27%)	54 (28%)	43 (28%)
	Mean (s.d.)	0.99 (0.92)	0.81 (0.92)	0.88 (0.92)	0.93 (0.87)	0.92 (0.86)
	Skew	0.55	(0.74)	0.64	0.39	0.41
**Non-core symptoms**						
Fatigue/loss of energy	Base rate (%)	317 (65%)	154 (51%)	134 (59%)	86 (45%)	81 (53%)
	Mean (s.d.)	1.69 (0.81)	1.43 (0.88)	1.62 (0.85)	1.44 (0.86)	1.52 (0.78)
	Skew	−0.39	−0.15	−0.20	0.12	−0.11
Appetite/weight change						
Appetite increase	Base rate (%)	90 (18%)	47 (16%)	37 (16%)	20 (11%)	17 (11%)
	Mean (s.d.)	1.03 (1.13)	0.81 (1.06)	0.86 (1.02)	0.73 (1.00)	0.70 (0.97)
	Skew	0.71	1.04	0.97	1.34	1.37
Appetite decrease	Base rate (%)	55 (11%)	27 (9%)	10 (4%)	14 (7%)	9 (6%)
	Mean (s.d.)	0.77 (0.83)	0.62 (0.78)	0.52 (0.69)	0.55 (0.75)	0.47 (0.74)
	Skew	0.87	1.16	1.36	1.28	1.68
Weight increase	Base rate (%)	73 (15%)	43 (14%)	46 (20%)	24 (13%)	30 (20%)
	Mean (s.d.)	0.74 (0.93)	0.67 (0.92)	0.85 (0.97)	0.67 (0.92)	0.83 (1.05)
	Skew	1.00	1.17	0.79	1.26	0.92
Weight decrease	Base rate (%)	77 (16%)	34 (11%)	23 (10%)	20 (11%)	19 (12%)
	Mean (s.d.)	0.77 (1.03)	0.58 (0.90)	0.61 (0.96)	0.57 (0.87)	0.62 (0.91)
	Skew	1.03	1.41	1.42	1.40	1.25
Sleep disturbance						
Insomnia	Base rate (%)	173 (35%)	88 (29%)	76 (33%)	69 (36%)	51 (33%)
	Mean (s.d.)	1.25 (0.77)	1.14 (0.78)	1.27 (0.81)	1.26 (0.73)	1.24 (0.75)
	Skew	0.34	0.52	0.38	0.31	0.29
Hypersomnia	Base rate (%)	75 (15%)	43 (14%)	27 (12%)	19 (10%)	20 (13%)
	Mean (s.d.)	0.69 (0.85)	0.65 (0.87)	0.59 (0.82)	0.52 (0.79)	0.66 (0.82)
	Skew	1.13	1.29	1.42	0.31	1.22
Psychomotor disturbance						
Psychomotor agitation	Base rate (%)	203 (41%)	107 (35%)	88 (38%)	56 (30%)	44 (29%)
	Mean (s.d.)	1.20 (0.91)	1.06 (0.94)	1.12 (0.95)	0.90 (0.89)	0.88 (0.84)
	Skew	0.07	0.30	0.22	0.43	0.30
Psychomotor retardation	Base rate (%)	184 (38%)	96 (32%)	81 (35%)	65 (34%)	51 (33%)
	Mean (s.d.)	1.06 (0.97)	0.91 (0.98)	1.02 (0.97)	0.94 (0.98)	0.91 (0.90)
	Skew	0.29	0.56	0.37	0.46	0.29
Worthlessness/guilt	Base rate (%)	226 (46%)	142 (47%)	95 (42%)	85 (45%)	69 (45%)
	Mean (s.d.)	1.67 (1.17)	1.62 (1.22)	1.61 (1.14)	1.63 (1.21)	1.62 (1.21)
	Skew	−0.03	−0.04	0.12	−0.01	0.02
Concentration difficulties	Base rate (%)	239 (49%)	122 (40%)	96 (42%)	70 (37%)	63 (41%)
	Mean (s.d.)	1.55 (0.83)	1.40 (0.83)	1.40 (0.83)	1.33 (0.79)	1.37 (0.79)
	Skew	0.16	0.29	0.22	0.33	0.20
Suicidal ideation	Base rate (%)	102 (21%)	50 (17%)	45 (20%)	35 (18%)	34 (22%)
	Mean (s.d.)	0.80 (0.85)	0.68 (0.77)	0.72 (0.83)	0.71 (0.78)	0.85 (0.82)
	Skew	0.72	0.75	0.79	0.62	0.57

IDS, Inventory of Depressive Symptomatology.

a. The base rate indicates the number of people scoring above the diagnostic threshold based on the Likert scale rating: 0 and 1 scored as 0 (symptom not present); 2 and 3 scored as 1 (symptom present).

### Statistical analysis

Cohen's kappa coefficients^22^ and percentage of agreement were used to assess the stability of the presence of individual diagnostic criterion symptoms across episodes. Spearman's correlation coefficients were calculated to estimate the associations between severity of symptoms across episodes, and Wilcoxon signed-rank tests were used to assess change in symptom severity between episodes. Linear regression analyses were performed to assess whether symptoms expressed during the baseline (index) episode predicted symptoms at recurrence. To assess the suitability of the data for linear regression, we calculated skewness and kurtosis to examine normality and produced scatterplots to examine homoscedasticity. Skewness and kurtosis were within ±2 for all symptoms, and scatterplots presented values that were approximately equally spaced along the *x*- and *y*-axes, indicating appropriate fit by the data for linear statistics. Significance was determined at α = 0.05/10 = 0.005, based on the number of comparisons performed per symptom, per analysis (Table 2).

**Table 2:** Cohen's kappa coefficients and percentage of agreement between major depressive disorder symptom presentation at baseline and at episode recurrence at 2-, 4-, 6- and 9-year follow-up^a^

IDS symptom		2 years (*n* = 302)	4 years (*n* = 229)	6 years (*n* = 190)	9 years (*n* = 154)
**Core symptoms**					
Depressed mood	Baseline	0.28 (63%)	0.28 (65%)	0.22 (61%)	0.23 (62%)
	2 years	−	0.33 (68%)	0.14 (59%)	0.16 (59%)
	4 years	−	−	0.21 (63%)	0.26 (63%)
	6 years	−	−	−	0.45 (73%)
Anhedonia	Baseline	0.38 (74%)	0.32 (71%)	0.34 (71%)	0.22 (70%)
	2 years	−	0.37 (72%)	0.32 (67%)	0.16 (64%)
	4 years	−	−	0.40 (73%)	0.46 (78%)
	6 years	−	−	−	0.42 (73%)
**Non-core symptoms**					
Fatigue/loss of energy	Baseline	0.32 (66%)	0.23 (64%)	0.28 (63%)	0.25 (63%)
	2 years	−	0.35 (69%)	0.36 (68%)	0.23 (62%)
	4 years	−	−	0.37 (69%)	0.18 (61%)
	6 years	−	−	−	0.39 (70%)
Appetite/weight change					
Appetite increase	Baseline	0.41 (77%)	0.40 (76%)	0.19 (68%)	0.20 (69%)
	2 years	−	0.26 (72%)	0.40 (82%)	0.29 (75%)
	4 years	−	−	0.38 (81%)	0.13 (68%)
	6 years	−	−	−	0.07 (79%)
Appetite decrease	Baseline	0.34 (80%)	0.31 (83%)	0.24 (77%)	0.28 (82%)
	2 years	−	0.22 (86%)	0.35 (83%)	0.25 (79%)
	4 years	−	−	0.07 (86%)	0.10 (81%)
	6 years	−	−	−	0.52 (91%)
Weight increase	Baseline	0.26 (74%)	0.18 (67%)	0.21 (76%)	0.10 (68%)
	2 years	−	0.25 (73%)	0.21 (80%)	0.15 (72%)
	4 years	−	−	0.42 (82%)	0.27 (79%)
	6 years	−	−	−	0.22 (71%)
Weight decrease	Baseline	0.19 (70%)	0.08 (71%)	0.09 (70%)	0.10 (75%)
	2 years	−	0.13 (79%)	0.13 (80%)	0.11 (77%)
	4 years	−	−	0.07 (80%)	0.15 (83%)
	6 years	−	−	−	0.06 (76%)
Sleep disturbance					
Insomnia	Baseline	0.41 (74%)	0.45 (74%)	0.41 (73%)	0.37 (72%)
	2 years	−	0.47 (75%)	0.43 (75%)	0.38 (72%)
	4 years	−	−	0.43 (74%)	0.31 (68%)
	6 years	−	−	−	0.35 (70%)
Hypersomnia	Baseline	0.34 (83%)	0.36 (84%)	0.38 (85%)	0.14 (80%)
	2 years	−	0.53 (88%)	0.43 (87%)	0.45 (87%)
	4 years	−	−	0.31 (87%)	0.34 (85%)
	6 years	−	−	−	0.58 (91%)
Psychomotor disturbance					
Psychomotor agitation	Baseline	0.40 (71%)	0.14 (58%)	0.20 (70%)	0.35 (66%)
	2 years	−	0.25 (65%)	0.50 (78%)	0.27 (69%)
	4 years	−	−	0.33 (70%)	0.16 (62%)
	6 years	−	−	−	0.22 (67%)
Psychomotor retardation	Baseline	0.40 (71%)	0.33 (69%)	0.34 (62%)	0.25 (71%)
	2 years	−	0.33 (70%)	0.37 (70%)	0.49 (78%)
	4 years	−	−	0.41 (72%)	0.39 (71%)
	6 years	−	−	−	0.41 (71%)
Worthlessness/guilt	Baseline	0.34 (67%)	0.35 (67%)	0.31 (65%)	0.32 (66%)
	2 years	−	0.25 (62%)	0.39 (70%)	0.37 (69%)
	4 years	−	−	0.42 (71%)	0.47 (73%)
	6 years	−	−	−	0.49 (75%)
Concentration difficulties	Baseline	0.33 (66%)	0.23 (61%)	0.29 (65%)	0.26 (64%)
	2 years	−	0.31 (66%)	0.16 (58%)	0.08 (54%)
	4 years	−	−	0.53 (77%)	0.21 (61%)
	6 years	−	−	−	0.43 (73%)
Suicidal ideation	Baseline	0.38 (80%)	0.32 (77%)	0.28 (77%)	0.42 (80%)
	2 years	−	0.38 (79%)	0.49 (85%)	0.32 (78%)
	4 years	−	−	0.39 (80%)	0.44 (80%)
	6 years	−	−	−	0.48 (79%)

IDS, Inventory of Depressive Symptomatology.

a. IDS Likert scale ratings were recoded: 0 and 1 scored as 0 (symptom not present); 2 and 3 scored as 1 (symptom present). Kappa coefficients <0.20 indicate poor consistency, 0.21–0.40 fair consistency, 0.41–0.60 moderate consistency, 0.61–0.80 good consistency and >0.81 very good consistency.^22^

## Results

### Sample characteristics

At baseline, *n* = 1115 participants met criteria for current (past 6 months) MDD (based on the CIDI), of whom *n* = 1027 still reported at least mild depression (IDS ≥ 14) at the time of assessment. Of those, *n* = 490 had a recurrent episode during at least one of the follow-ups during the 9 years. Those who experienced recurrence during follow-up did not differ from those who did not in gender (χ^2^(1, *n* = 1027) = 0.38, *P* = 0.549) or MDD history (χ^2^(1, *n* = 1027) = 0.05, *P* = 0.849), but were older (*t*(1025) = −4.29, *P* < 0.001, *d* = 0.27) and more likely to have a lifetime history of an anxiety disorder (χ^2^(1, *n* = 1027) = 7.39, *P* = 0.007).

At baseline, the sample (*n* = 490) was 67% female, with a mean age of 42.8 years (range 18–64, s.d. = 11.9). At 2-year follow-up, *n* = 302 (64%) met criteria for current MDD, at 4 years *n* = 229 (47%), 6 years *n* = 190 (39%) and 9 years *n* = 154 (31%). Mean (overall) depression severity (IDS) at baseline was 35.8 (s.d. = 10.7), at 2-year follow-up 32.4 (s.d. = 10.6), 4 years 34.6 (s.d. = 11.0), 6 years 32.9 (s.d. = 10.9) and 9 years 34.1 (s.d. = 10.3). Compared with baseline, symptom severity was lower at episode recurrence at 2 years (*t*(301) = 9.48, *P* < 0.001, *d* = 0.55), 4 years (*t*(228) = 4.26, *P* < 0.001, *d* = 0.28), 6 years (*t*(189) = 4.57, *P* < 0.001, *d* = 0.33) and 9 years (*t*(153) = 2.01, *P* = 0.023, *d* = 0.16). Symptom severity at baseline was significantly correlated with severity at episode recurrence (2 years: *r* = 0.58, *P* < 0.001; 4 years: *r* = 0.51, *P* < 0.001; 6 years: *r* = 0.52, *P* < 0.001; 9 years: *r* = 0.54, *P* < 0.001).

### Stability of symptoms across recurrent episodes (IDS)

Cohen's kappa coefficients and percentage of agreement represent stability of symptom presentation across recurrent episodes based on the IDS (Table 2). Based on kappa, stability of symptom presentation throughout multiple recurrent episodes was highest for insomnia, worthlessness/guilt and suicidal ideation (i.e. these symptoms exhibited fair-to-moderate consistency across all episodes).

### Stability of symptom severity across recurrent episodes (IDS)

Spearman's correlation coefficients represent the stability of symptom severity across recurrent episodes based on the IDS (Table 3). Throughout multiple episodes, appetite increase, insomnia and hypersomnia, psychomotor retardation, worthlessness/guilt and suicidal ideation retained the highest relative stability (i.e. exhibited medium-to-large correlations across all episodes). Insomnia had the most stability as the only symptom to exhibit large correlations across all episodes.

**Table 3:** Spearman correlations between major depressive disorder symptom severity at baseline and at episode recurrence at 2-, 4-, 6- and 9-year follow-up^a^

IDS symptom		2 years (*n* = 302)	4 years (*n* = 229)	6 years (*n* = 190)	9 years (*n* = 154)
**Core symptoms**					
Depressed mood	Baseline	**0.34**	**0.34**	**0.28**	**0.32**
	2 years	−	**0.43**	**0.29**	**0.31**
	4 years	−	−	0.25	0.26
	6 years	−	−	−	**0.56**
Anhedonia	Baseline	**0.39**	**0.39**	**0.36**	**0.26**
	2 years	−	**0.45**	**0.45**	0.24
	4 years	−	−	**0.45**	**0.44**
	6 years	−	−	−	**0.58**
**Non-core symptoms**					
Fatigue/loss of energy	Baseline	**0.39**	**0.32**	**0.28**	0.21
	2 years	−	**0.45**	**0.41**	0.26
	4 years	−	−	**0.51**	**0.32**
	6 years	−	−	−	**0.40**
Appetite/weight change					
Appetite increase	Baseline	**0.55**	**0.47**	**0.31**	**0.49**
	2 years	−	**0.60**	**0.43**	**0.44**
	4 years	−	−	**0.46**	**0.46**
	6 years	−	−	−	0.42
Appetite decrease	Baseline	**0.42**	**0.28**	0.28	0.32
	2 years	−	**0.42**	0.23	0.16
	4 years	−	−	0.17	0.07
	6 years	−	−	−	0.45
Weight increase	Baseline	**0.25**	**0.23**	.27	0.30
	2 years	−	**0.34**	**0.35**	0.33
	4 years	−	−	**0.44**	0.26
	6 years	−	−	−	0.42
Weight decrease	Baseline	**0.24**	0.09	0.20	0.13
	2 years	−	0.03	0.36	0.07
	4 years	−	−	0.28	0.33
	6 years	−	−	−	0.15
Sleep disturbance					
Insomnia	Baseline	**0.56**	**0.59**	**0.59**	**0.60**
	2 years	−	**0.66**	**0.69**	**0.55**
	4 years	−	−	**0.64**	**0.56**
	6 years	−	−	−	**0.65**
Hypersomnia	Baseline	**0.48**	**0.53**	**0.50**	**0.35**
	2 years	−	**0.51**	**0.55**	**0.35**
	4 years	−	−	**0.60**	**0.47**
	6 years	−	−	−	**0.57**
Psychomotor disturbance					
Psychomotor agitation	Baseline	**0.48**	**0.21**	**0.43**	**0.26**
	2 years	−	**0.37**	**0.56**	0.28
	4 years	−	−	**0.46**	0.31
	6 years	−	−	−	0.31
Psychomotor retardation	Baseline	**0.41**	**0.42**	**0.29**	**0.37**
	2 years	−	0.41	0.51	0.46
	4 years	−	.	**0.59**	**0.42**
	6 years	−	−	−	**0.45**
Worthlessness/guilt	Baseline	**0.40**	**0.37**	**0.34**	**0.31**
	2 years	−	**0.40**	**0.42**	**0.38**
	4 years	−	−	**0.47**	**0.47**
	6 years	−	−	−	**0.45**
Concentration difficulties	Baseline	**0.40**	**0.26**	**0.24**	**0.28**
	2 years	−	**0.29**	0.24	0.10
	4 years	−	−	**0.52**	0.17
	6 years	−	−	−	**0.45**
Suicidal ideation	Baseline	**0.46**	**0.48**	**0.36**	**0.48**
	2 years	−	**0.49**	**0.53**	**0.39**
	4 years	−	−	**0.39**	**0.45**
	6 years	−	−	−	**0.55**

IDS, Inventory of Depressive Symptomatology

a. Interpretation of correlation coefficients based on *r* = 0.50 indicating large, *r* = 0.30 medium, and *r* = 0.10 small correlations.^23^ Significant correlations based on *P* < 0.005 are indicated in bold.

### Change in symptom severity across recurrent episodes (IDS)

Compared with baseline, symptoms were significantly lower at episode recurrence at 2 years for depressed mood (*Z* = ***−***5.03, *P* < 0.001), anhedonia (*Z* = ***−***4.80, *P* < 0.001), fatigue (*Z* = ***−***6.19, *P* < 0.001), appetite increase (*Z* = ***−***2.97, *P* = 0.003) and decrease (*Z* = ***−***2.97, *P* = 0.003), insomnia (*Z* = ***−***3.45, *P* = 0.001), psychomotor agitation (*Z* = ***−***4.00, *P* < 0.001) and retardation (*Z* = ***−***4.37, *P* < 0.001), concentration difficulties (*Z* = ***−***4.01, *P* < 0.001) and suicidal ideation (*Z* = ***−***3.66, *P* < 0.001), but not for weight increase (*P* = 0.302) or decrease (*P* = 0.022), hypersomnia (*P* = 0.784) or worthlessness/guilt (*P* = 0.048). At 4 years, only anhedonia (*Z* = ***−***3.69, *P* < 0.001) and concentration difficulties (*Z* = ***−***3.37, *P* = 0.001) were significantly lower than at baseline (for all other symptoms, *P* ≥ 0.005). At 6 years, symptom severity was lower for anhedonia (*Z* = ***−***2.96, *P* = 0.003), fatigue (*Z* = ***−***3.74, *P* < 0.001), hypersomnia (*Z* = ***−***2.99, *P* = 0.003), psychomotor agitation (*Z* = ***−***3.31, *P* = 0.001) and concentration difficulties (*Z* = ***−***2.95, *P* = 0.003) (for all other symptoms, *P* ≥ 0.005). At 9 years, symptom severity compared with baseline was lower only for appetite increase (*Z* = ***−***4.22, *P* < 0.001) and psychomotor agitation (*Z* = ***−***2.92, *P* = 0.003) (for all other symptoms, *P* ≥ 0.005).

### Predicting symptom severity across recurrent episodes (IDS)

Linear regression results predicting symptom severity from baseline episode to episode recurrence are presented in Table 4. Baseline symptom severity consistently significantly predicted symptoms at all subsequent episodes for appetite increase, insomnia, hypersomnia, psychomotor retardation and agitation, worthlessness/guilt, concentration difficulties and suicidal ideation. The largest effect sizes were observed for insomnia (*R*^2^ ≥ 0.32).

**Table 4:** Linear regression of baseline symptom severity predicting symptoms at episode recurrence at 2-, 4-, 6- and 9-year follow-up

IDS symptom	Follow-up	*B*	s.e.	*t*	*P*^a^	*R*^2^
**Core symptoms**						
Depressed mood	2 years	0.32	0.05	6.34	**<0.001**	0.12
	4 years	0.31	0.06	5.32	**<0.001**	0.11
	6 years	0.27	0.06	4.15	**<0.001**	0.08
	9 years	0.34	0.08	4.37	**<0.001**	0.11
Anhedonia	2 years	0.41	0.05	8.82	**<0.001**	0.19
	4 years	0.38	0.06	6.53	**<0.001**	0.16
	6 years	0.34	0.06	5.74	**<0.001**	0.15
	9 years	0.27	0.08	3.44	**0.001**	0.07
**Non-core symptoms**						
Fatigue/loss of energy	2 years	0.43	0.06	7.41	**<0.001**	0.16
	4 years	0.36	0.07	5.05	**<0.001**	0.10
	6 years	0.28	0.07	3.77	**<0.001**	0.07
	9 years	0.17	0.08	2.15	0.033	0.03
Appetite/weight change						
Appetite increase	2 years	0.50	0.06	7.92	**<0.001**	0.30
	4 years	0.43	0.08	5.43	**<0.001**	0.20
	6 years	0.29	0.09	3.19	**0.002**	0.09
	9 years	0.36	0.07	4.95	**<0.001**	0.23
Appetite decrease	2 years	0.42	0.08	3.65	**<0.001**	0.20
	4 years	0.26	0.08	3.32	**0.001**	0.09
	6 years	0.24	0.10	2.46	0.016	0.06
	9 years	0.39	0.11	3.51	**0.001**	0.15
Weight increase	2 years	0.25	0.07	3.48	**0.001**	0.07
	4 years	0.43	0.07	2.65	0.009	0.05
	6 years	0.23	0.10	2.37	0.020	0.05
	9 years	0.33	0.12	2.76	0.007	0.09
Weight decrease	2 years	0.18	0.07	2.49	0.014	0.04
	4 years	0.11	0.10	1.11	0.268	0.01
	6 years	0.16	0.09	1.83	0.071	0.04
	9 years	0.13	0.12	1.12	0.267	0.02
Sleep disturbance						
Insomnia	2 years	0.56	0.05	11.96	**<0.001**	0.32
	4 years	0.60	0.06	10.88	**<0.001**	0.34
	6 years	0.54	0.06	9.44	**<0.001**	0.32
	9 years	0.61	0.07	9.45	**<0.001**	0.37
Hypersomnia	2 years	0.46	0.05	8.51	**<0.001**	0.20
	4 years	0.45	0.05	8.40	**<0.001**	0.24
	6 years	0.44	0.06	7.45	**<0.001**	0.23
	9 years	0.35	0.08	4.30	**<0.001**	0.11
Psychomotor disturbance						
Psychomotor agitation	2 years	0.50	0.05	9.47	**<0.001**	0.23
	4 years	0.24	0.07	3.60	**<0.001**	0.05
	6 years	0.38	0.06	6.12	**<0.001**	0.17
	9 years	0.23	0.07	3.20	**0.002**	0.06
Psychomotor retardation	2 years	0.42	0.05	7.95	**<0.001**	0.18
	4 years	0.41	0.06	6.83	**<0.001**	0.17
	6 years	0.30	0.07	4.07	**<0.001**	0.08
	9 years	0.36	0.07	5.08	**<0.001**	0.15
Worthlessness/guilt	2 years	0.42	0.06	7.50	**<0.001**	0.16
	4 years	0.36	0.06	5.87	**<0.001**	0.13
	6 years	0.35	0.07	4.92	**<0.001**	0.12
	9 years	0.31	0.08	4.02	**<0.001**	0.10
Concentration difficulties	2 years	0.39	0.05	7.48	**<0.001**	0.16
	4 years	0.25	0.06	3.99	**<0.001**	0.07
	6 years	0.20	0.07	2.88	**0.004**	0.04
	9 years	0.27	0.08	3.53	**0.001**	0.08
Suicidal ideation	2 years	0.40	0.05	8.49	**<0.001**	0.20
	4 years	0.44	0.06	7.53	**<0.001**	0.20
	6 years	0.34	0.06	5.28	**<0.001**	0.13
	9 years	0.48	0.07	6.85	**<0.001**	0.24

IDS, Inventory of Depressive Symptomatology.

a. Significant associations based on *P* < 0.005 are indicated in bold.

## Discussion

To better understand the stability of depressive symptoms across episodes, we examined symptom presentation and severity in recurrent major depression over 9 years. Overall, we observed low concordance in symptoms across episodes. The most evidence was found for the stability of insomnia, worthlessness/guilt and suicidal ideation over time. Contrary to prior reports, however, suicidal ideation did not emerge as uniquely stable in comparison with other non-core symptoms.

### Consistency in symptom presentation across episodes

We first examined consistency in symptom presentation over time, i.e. whether symptoms reached diagnostic threshold across multiple episodes. Kappa estimates indicated low consistency in symptom presentation overall, with most symptoms reaching only fair consistency across episodes. This indicates that patients do not consistently present with the same symptoms across recurrent episodes. Estimates for insomnia, worthlessness/guilt and suicidal ideation maintained fair-to-moderate consistency across all episodes (reaching moderate consistency across some, but not all, episodes). These symptoms had the highest relative stability over time, although still reflecting only modest stability in absolute terms. This is in line with previous studies indicating little stability in symptoms across episodes^4–7^ and that symptoms (as defined by diagnostic criteria in DSM-5^17^) experienced during a certain episode may not reach the diagnostic threshold during a subsequent episode.^4,6^

### Stability in the severity of symptoms across episodes

We then examined stability in the severity of symptoms and, overall, observed moderate consistency across episodes (i.e. medium correlations across episodes for most symptoms). Appetite increase, insomnia, psychomotor retardation, worthlessness/guilt and suicidal ideation retained the highest relative stability over time by exhibiting medium-to-large correlations across episodes, with insomnia being the only symptom that maintained large correlations across all episodes. Of note here is that our categorisation of the coefficients as representing small, medium and large correlations, based on Cohen's guidelines,^23^ is fairly lenient, as many others (e.g.^24^) would consider only coefficients ≥0.70 as representing large correlations – a threshold none of our estimates reached. In accordance, prior research also indicates that stability in symptom severity across episodes tends to be fairly low, although most studies have focused on overall symptom severity (e.g.^4,25,26^), rather than stability in the severity of individual symptoms.^7,11^

### Baseline symptom severity as a predictor of severity at recurrence

Finally, we found IDS symptom severity at the baseline (index) episode to significantly predict symptoms at recurrence across 9 years for insomnia, hypersomnia, psychomotor retardation and agitation, worthlessness/guilt, concentration difficulties and suicidal ideation. Hence, despite little symptom stability, it appears that symptom severity during a previous depressive episode is still a significant indicator of symptom severity at recurrence, at least for the subset of symptoms exhibiting greater than average stability.

### Comparison with the literature: insomnia, worthlessness/guilt, suicidal ideation

Taken together, insomnia, worthlessness/guilt and suicidal ideation were the most stable symptoms across episodes. Of these, insomnia had the greatest relative stability (as indicated by the highest correlations across episodes). This finding is in line with Gosek and colleagues,^13^ who found insomnia (together with concentration difficulties) to be the most consistent symptom in recurrent depression after retrospectively reviewing medical records of 29 individuals with MDD. Indeed, insomnia is a common and often persistent disturbance which affects up to one-third of the general population,^27,28^ and hence a symptom that may persist not only during but also between episodes. Insomnia has also been found to prospectively predict the onset of depression.^29,30^ Further, few people with insomnia seek professional help,^28^ and sleep disturbances may receive less attention during depression treatment, which can lead to their chronicity. Prior research has also highlighted worthlessness as one of the most common depressive symptoms, reported by up to 90% of depressed patients.^31^ Another study showed low self-esteem to predict both higher residual symptoms and increased risk of recurrence after an initial depressive episode.^32^ A negative self-image (encompassing a sense of worthlessness) may represent a more enduring cognitive schema,^33^ which would explain its stability over time. Likewise, suicidal ideation reactivity (i.e. the tendency for suicidal cognitions to become activated in response to reductions in mood) remains stable also in remission.^34^ Further, suicidal ideation may also exist in the absence of a depressive disorder. Hence suicidal ideation can be considered a distinct construct,^35^ with significant overlap especially with more severe depression,^36^ rather than simply a symptom of depression. Taken all together, disturbances that are more likely to persist between episodes would logically also show the highest inter-episode stability. However, it remains to be examined whether worthlessness/guilt, suicidal ideation and insomnia are indeed more likely than other depressive symptoms to persist at times of remission, as in the present study we specifically examined symptoms within recurrent episodes, and did not examine residual symptoms at time points when participants did not meet diagnostic criteria.

Contrary to previous reports,^7,11,12^ however, we did not observe suicidal ideation to be uniquely stable. Although suicidal ideation did exhibit fair-to-moderate consistency and medium-to-large correlations across episodes, these estimates were only marginally higher than those for most other symptoms. Explanations for our non-replication may reflect differences in study design and sample size. First, we assessed symptoms over 9 years and up to five episodes, whereas previous studies have focused on a maximum time frame of up to 2 years. A higher stability of suicidal ideation was found across 12 months^11^ than across 24 months.^7^ Unsurprisingly, symptom stability is generally higher between two consecutive episodes occurring closer in time.^37,38^ Further, the two studies highlighting the stability of suicidal ideation used relatively small samples (*n* < 70 in both studies).^7,11^

### Consistency in core symptoms

Although we specifically focused on examining consistency in non-core symptoms, with the assumption that core symptoms would by nature exhibit more consistency and not be comparable to non-core symptoms, it is noteworthy that we did not observe a high level of consistency in the two core symptoms either. Instead, consistency in these symptoms was comparable to, or somewhat lower than, that of the most stable non-core symptoms (i.e. insomnia, worthlessness/guilt and suicidal ideation). This indicates that patients may also not consistently present with the same core symptom across episodes, or at least not to the same degree of severity. This is also in line with Oquendo and colleagues,^7^ who found the severity of depressed mood to have one of the lowest correlations across episodes. On the contrary, previous studies have indicated high consistency for anhedonia, at least between two consecutive episodes.^7,13^

### Implications for research and clinical practice

Our findings support the notion that suicidal ideation is moderately stable in recurrent depression -however, it does not exhibit substantially more stability than most other non-core symptoms of depression. Instead, insomnia exhibited the highest stability, although differences between symptoms were often small in magnitude, and it is difficult to confidently state that insomnia would be meaningfully more stable than most other symptoms. It is conceivable that symptoms that have an increased risk of chronicity (such as suicidal ideation and insomnia) and may persist between episodes also have the highest consistency across episodes, but this requires further study. Due to the high recurrence rate of depressive disorders, understanding how recurrence looks is of great importance; whether patients are likely to present with the same symptoms across episodes has implications for both diagnosis and treatment thereafter. For example, a recurrent episode with a different symptom profile may signal different treatment needs. Further, understanding which symptoms form the most temporally stable components of the depressive syndrome is also highly relevant for relapse prevention.

### Limitations

Limitations of the present study include the reliance on only one depression measure (the IDS). Comparison of results from two or more instruments would provide a more comprehensive picture of symptom presentation. We also did not account for the use of medications or psychological treatments in our analyses. NESDA is a naturalistic cohort study and type and dosage of treatment are not standardised, and therefore accounting for the heterogeneity of different treatments and doses was not feasible. It is possible that receiving treatment during certain episodes may have led to lower stability estimates between corresponding time points. Further, attrition affects most longitudinal studies, and within NESDA, participants with worse symptom profiles have been shown to be at higher risk of dropping out.^39^ It is possible that those with more severe and/or chronic depression have different symptom stability profiles, and our findings may have more limited generalisability to these populations. However, with a mean baseline IDS score of 36, the severity of depression in our sample was moderate to severe. Consequently, regression to the mean is likely to have affected our sample at the follow-ups and potentially reduced stability estimates, as we observed significant reductions in the severity of most symptoms over time, especially between baseline and the first follow-up. Finally, the kappa statistic is affected by prevalence rates, which may lower stability estimates for symptoms with lower base rates (such as suicidal ideation). This may have reduced the kappa estimates for suicidal ideation in comparison with symptoms with higher prevalence rates, such as insomnia and worthlessness/guilt. However, this would not affect estimates of the stability of symptom severity (as characterised by Spearman correlations).

## Supporting information

## Data Availability

Data requests may be submitted through the NESDA website (www.nesda.nl).
